# A Rare Case of Diffuse Leptomeningeal and Cortical Enhancement Secondary to Stroke-Like Migraine Attacks After Radiation Therapy (SMART) Syndrome in a Patient With a History of Childhood Medulloblastoma

**DOI:** 10.7759/cureus.69435

**Published:** 2024-09-15

**Authors:** Christopher O Adereti, Joy E Burke, Jonathan R Pace

**Affiliations:** 1 Neurosurgery, Lahey Hospital and Medical Center, Burlington, USA; 2 Neurology, Lahey Hospital and Medical Center, Burlington, USA

**Keywords:** craniospinal irradiation, leptomeningeal disease, leptomeningeal enhancement, pediatric medulloblastoma, smart syndrome

## Abstract

Stroke-like migraine attacks after radiation therapy (SMART) syndrome is a rare complication of craniospinal irradiation (CSI). Patients commonly present with headaches, seizures, and paroxysmal focal neurological deficits. There is a dearth of studies reported in the literature with an estimated fewer than 100 cases described since it was initially defined in the mid-1990s. The authors present the case of a 23-year-old patient with a history of childhood medulloblastoma and prior ventriculoperitoneal shunt (VPS), chemotherapy, and CSI who presented with headaches and new-onset seizures. Magnetic resonance imaging (MRI) of the brain showed diffuse left temporoparietal and occipital leptomeningeal and cortical enhancement. However, cerebrospinal fluid (CSF) analysis was unremarkable for neoplastic, infectious, or inflammatory etiology. Initiation of systemic steroid therapy resulted in drastic improvement of the patient’s symptoms and prompted antiepileptic drug (AED) wean and persistent resolution of leptomeningeal and cortical contrast enhancement on brain MRI. When evaluating MRI evidence of leptomeningeal enhancement, neurosurgeons should consider SMART syndrome in the differential diagnosis, especially when extensive workup rules out more common causes of this finding such as leptomeningeal disease (LMD). Proper identification of SMART syndrome can lead to timely treatment, avoidance of invasive procedures such as tissue biopsy, and improved clinical outcomes.

## Introduction

Stroke-like migraine attacks after radiation therapy (SMART) syndrome is a rare and likely underreported late complication of brain irradiation. Patients commonly present with headaches, seizures, and paroxysmal focal neurological deficits [1-3]. The exact pathogenesis of this syndrome remains yet to be known, but studies suggest that it may involve pathways similar to migraines, epilepsy, and posterior reversible encephalopathy syndrome [3,4]. Less than 100 cases of SMART syndrome have been described in the literature since it was initially reported in 1995 [2,3,5,6]. We discuss the occurrence of SMART syndrome in a patient initially suspected to have leptomeningeal disease (LMD). We also briefly discuss associations between SMART syndrome and its variants.

## Case presentation

A 23-year-old female with a history of childhood medulloblastoma, prior ventriculoperitoneal shunt (VPS) placement, chemotherapy, and craniospinal irradiation (CSI) presented with headache and new-onset seizures. At age 12, the patient was initially diagnosed with multiple brain lesions (large lobulated masses involving the posterior fossa and suprasellar cistern) after developing progressive symptoms of headache, nausea and vomiting, abdominal pain, decreased appetite, and gait ataxia. After failing to improve with medical management, the patient underwent a suboccipital craniotomy with external ventricular drain (EVD) placement, and the pathology of the lesion showed a primitive neuroectodermal tumor consistent with medulloblastoma.

The patient's EVD was removed on postoperative day 5; however, subsequent development of non-communicating hydrocephalus over the ensuing days resulted in a need for VPS placement. She received adjuvant chemotherapy that included weekly vincristine (1.6 mg via a chemo port), with a subsequent taper to 0.79 mg weekly over a two-month duration. Over the course of the following year, she received eight additional cycles of cisplatin and lomustine and underwent a single round of CSI consisting of 55 Grays.

On arrival at our hospital, history was obtained from the patient and her mother. The patient reported having acute frontal headache four days before hospital admission with increased frequency and intensity the next day. Two days prior to admission, the patient experienced a first-time seizure event followed by a recurrent episode later that same day. These episodes were described as focal in nature, with staring spells. The next day, she experienced two to three generalized seizure events. The patient was then admitted to the neurology service where she was initiated on antiepileptic drug (AED) therapy consisting of levetiracetam (LEV) 750 mg BID and lacosamide (LCM) 150 mg BID and placed on continuous video electroencephalography monitoring while detailed workup was obtained.

On initial assessment, she appeared sleepy, although arousable and cooperative during the neurological exam. She did have some confusion and dysarthric speech, but otherwise she had good comprehension and repetition. The remainder of the exam was non-focal. Computed tomography of the head was obtained on hospital day (HOD) 1 and demonstrated stable postoperative changes from prior medulloblastoma resection and VPS placement but was otherwise unrevealing for stroke or other acute pathology.

**Figure 1:**
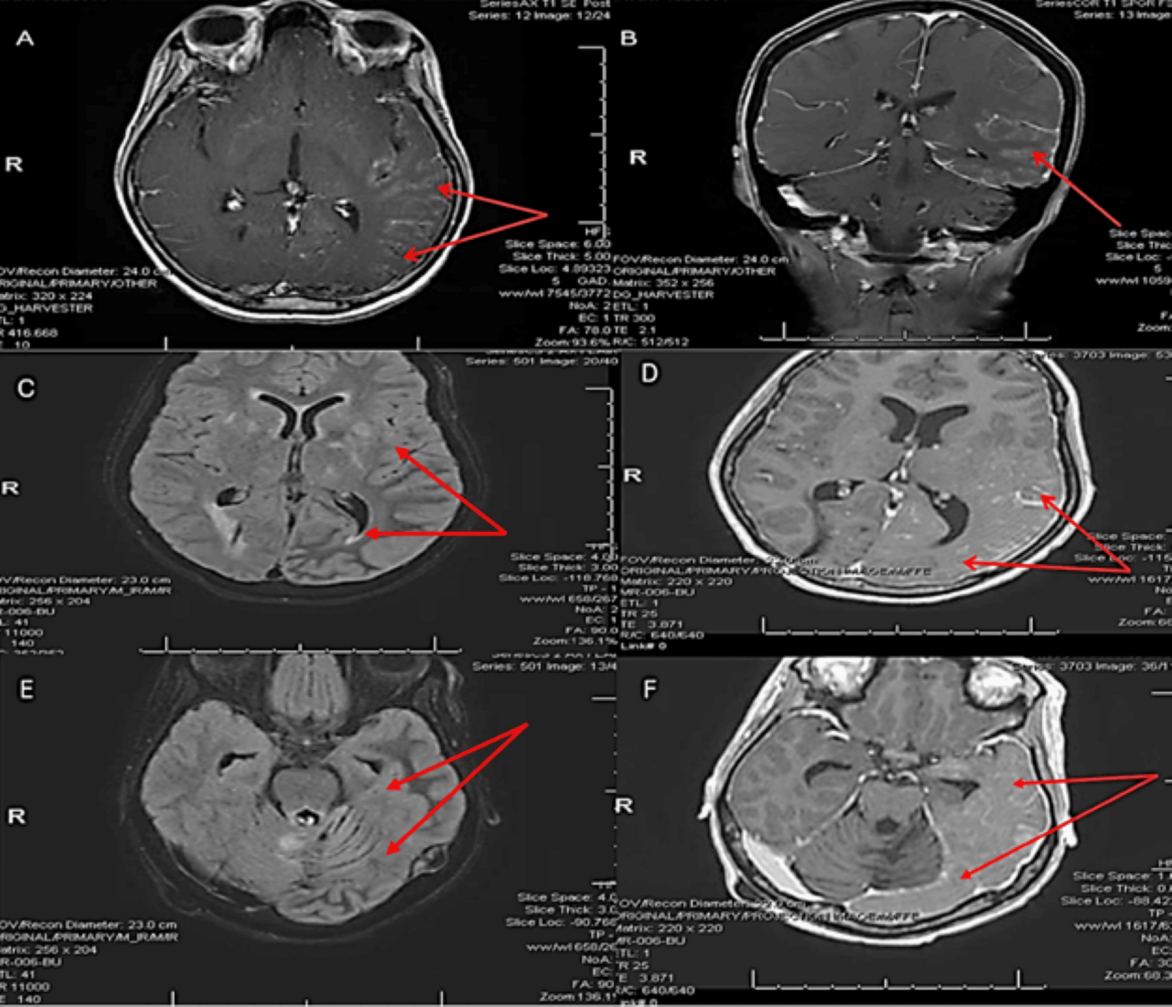
Axial T1-weighted SE sequence with contrast showing diffuse left temporoparietal and occipital leptomeningeal and cortical enhancement in the region of prior radiation therapy (red arrows) (A) and corresponding enhancement of the left temporal lobe as seen on coronal view (B). T1 FLAIR without contrast in the aforementioned regions (C and E) and their corresponding findings on T1-weighted sequence with contrast.

**Figure 2:**
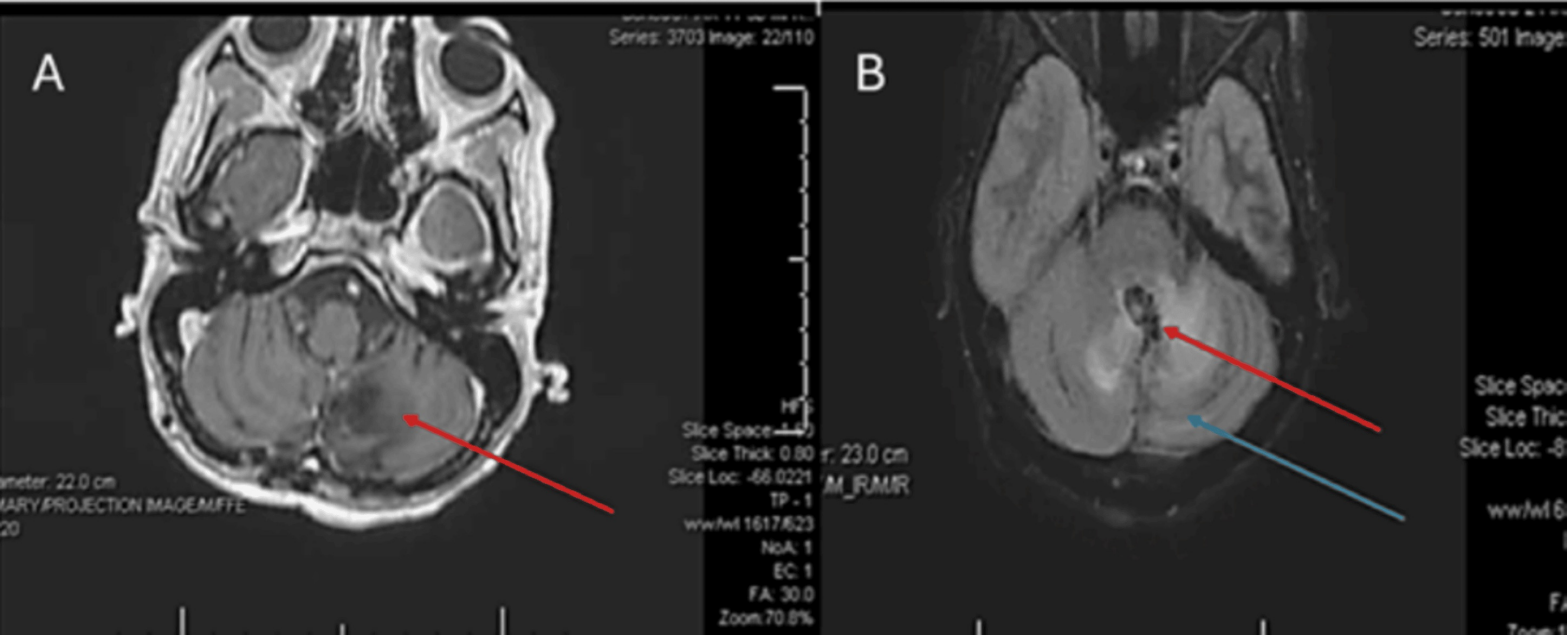
Contrast-enhanced axial T1-weighted sequence of the cerebellum (A) and corresponding T1 FLAIR sequence (B). The red arrows demonstrate expected post-surgical changes from the patient’s prior tumor removal. However, there is a notable absence of leptomeningeal enhancement in the cerebellar folia (blue arrow), which would be unusual in the setting of LMD. This further supports a diagnosis of SMART syndrome. LMD, leptomeningeal disease

Over the next four to five days, the patient experienced numerous clinical and electrographic focal seizures that then prompted adjustments to her AEDs: clobazam (CLB) 10 mg BID and oxcarbazepine (OXC) 300 mg BID were added while LEV was increased to 1 g BID. Contrast-enhanced T1-weighted magnetic resonance imaging (MRI) of the brain was obtained on HOD 4 and showed diffuse left temporoparietal and occipital leptomeningeal and cortical enhancement (best appreciated on axial and coronal imaging) (Figure 1) while regional edema was most notable on FLAIR sequence. There were expected postoperative changes in the cerebellum; however, there was a notable absence of abnormal leptomeningeal enhancement in the parenchyma (Figure 2).

**Figure 3:**
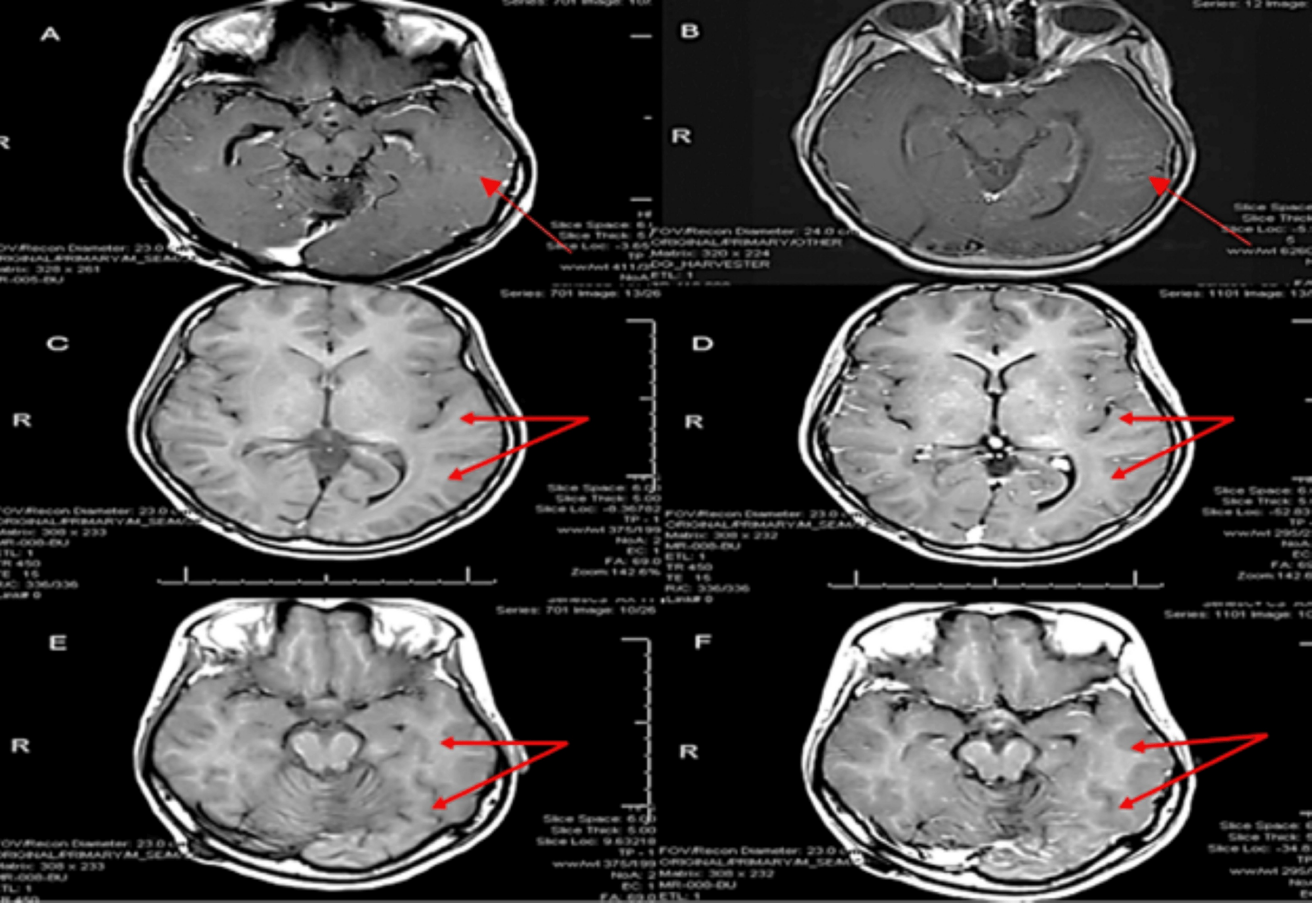
Axial T1-weighted sequence with contrast showing interval resolution of left temporoparietal occipital leptomeningeal and cortical enhancement (red arrows) (A) compared with that seen in the initial study three weeks prior (B). A repeat T1-weighted MRI without contrast (C and E) and with contrast (D and F) at the initial six-week follow-up appointment shows continued resolution of leptomeningeal and cortical enhancement. MRI, magnetic resonance imaging

Given the possibility of LMD in the setting of prior medulloblastoma and CSI, further workup was indicated. MRI of the cervical, thoracic, and lumbar spine was obtained and unremarkable for leptomeningeal enhancement. Infectious workup including blood cultures, complete blood count (Table 1), lactic acid, basic metabolic panel (Table 2), chest x-ray, and urinalysis was obtained and largely unremarkable. However, the patient's C-reactive protein was mildly elevated at 10 mg/L. Cerebrospinal fluid (CSF) analysis was negative for neoplastic, infectious, or inflammatory etiology (Table 3). Initiation of systemic pulse steroid therapy, intravenous (IV) methylprednisolone 1 g/daily for five-day duration, resulted in drastic improvement of the patient’s symptoms with seizure freedom. Furthermore, there was persistent resolution of leptomeningeal enhancement on brain MRI within three weeks from initial presentation and continuing through to her six-week post-hospital discharge appointment (Figure 3). Thus, this reassurance prompted an AED wean: CLB was reduced to 5 mg BID and OXC 300 mg BID was discontinued. However, she remained on LEV 1 g BID and LCM 150 mg BID.

**Table 1:** Initial complete blood count results along with their corresponding reference range and units upon hospital arrival. WBC, white blood cell count; RBC, red blood cell count; MCV, mean corpuscular volume; RDW, red-distribution width

Lab	Reference range and units	Panel result
WBC	4.00-11.00 K/uL	8.5
RBC	4.10-5.10 M/uL	3.83
Hemoglobin	12.0-15.3 g/dL	11.3
Hematocrit	36.0-45.0%	33.0
MCV	80-96 fL	86
RDW	11.6-14.6 %	12.4
Platelet count	150-450 K/uL	323

**Table 2:** Initial basic metabolic panel, magnesium, lactic acid, and C-reactive protein results along with their corresponding reference range and units upon hospital arrival. BUN, blood urea nitrogen; e-GFR, estimated glomerular filtration rate

Lab	Reference range and units	Panel result
Sodium	135-146 mmol/L	137
Potassium	3.5-5.2 mmol/L	3.5
Chloride	98-110 mmol/L	103
Bicarbonate	24-32 mmol/L	20
Anion gap	2-15 mmol/L	14
BUN	7- 24 mg/dL	5
Creatinine	0.5-1.1 mg/dL	0.7
Glucose	70-118 mg/dL	97
Calcium	8.5-10.5 mg/dL	8.9
e-GFR	≥60 mL/min/BSA	>120
Magnesium	1.6-2.6 mg/dL	1.6
Lactic acid	0.5-2.0 mmol/L	1.3
C-reactive protein	<5.0 mg/L	10.2

**Table 3:** CSF cytology results along with corresponding reference range and units. Panel result 1 displays initial results from studies obtained three days after the patient’s hospital arrival. Panel result 2 displays results from a repeat lumbar puncture obtained nine days later. Results were negative for neoplastic, infectious, or inflammatory etiology. *Cell count 2 (only two tubes for analysis) **Lab not reordered on repeat CSF analysis CSF, cerebrospinal fluid; Ab, antibody; IFA, immunofluorescence assay; STS, serological test for syphilis; VDRL, venereal disease research laboratory test; N/A, not available; PCR, polymerase chain reaction

Lab	Reference range and units	Panel result 1	Panel result 2
CSF cell count 1	N/A	50	108
CSF cell count 4	N/A	45*	45
Glucose, CSF	40-70 mg/dL	86 (H)	129 (H)
Total protein, CSF	15-45 mg/dL	174 (H)	129 (H)
Lactate dehydrogenase, CSF	<39 U/L	100 (H)	**
Angiotensin-converting enzyme, CSF	≤15 U/L	6	**
Protein electrophoresis, CSF	N/A	Repeat	**
Paraneoplastic Ab screen, IFA, CSF	N/A	Repeat	**
STS (VDRL), CSF	Nonreactive	Nonreactive	**
Herpes simplex virus PCR	N/A	Not detected	**
Varicella zoster virus PCR	N/A	Not detected	**

## Discussion

Patients with a history of brain radiotherapy can experience acute stroke-like syndromes related to the delayed effects of brain irradiation, including SMART syndrome, peri-ictal pseudoprogression (PIPG), and acute late-onset encephalopathy after radiation therapy syndrome (ALERT) syndrome [5,7,8]. The exact pathophysiology of SMART syndrome is unknown but hypothesized to be multifactorial. Delayed cranial irradiation injury involves white matter necrosis, vascular endothelial damage, demyelination, and gliosis [9]. However, there is little histologic evidence of SMART syndrome [2,10]. Furthermore, radiation-induced mitochondrial dysfunction has been implicated in SMART syndrome pathophysiology [11-13].

The diagnosis of SMART syndrome is challenging because clinical symptoms and imaging features of SMART syndrome are indeterminate and overlap with tumor recurrence and other neurologic diseases, which may result in inappropriate clinical management and unnecessary invasive diagnostic procedures [11]. Black et al. proposed diagnostic criteria in 2006, including 1) a remote history of external beam cranial irradiation; 2) prolonged, reversible signs and symptoms referable to a unilateral cortical region beginning years after cranial irradiation, including seizure, migraine with or without an aura, and stroke-like symptoms; 3) transient, diffuse, unilateral gyriform enhancement sparing the white matter within a previous radiation field; and 4) not attributed to another disorder [14].

However, since this proposal, subsequent cases of SMART syndrome have revealed additional clinical and imaging manifestations that do not firmly adhere to these criteria despite otherwise matching the features of SMART syndrome [11]. Updates regarding clinical and imaging prognostic factors and a spectrum of SMART syndrome variants including PIPG and ALERT syndrome have been proposed [5,7,8,11]. Unlike SMART syndrome, PIPG can be observed in all cortical regions, while the former occurs predominantly in the temporal, parietal, and occipital lobes. A link between steroid therapy and clinical improvement has yet to be established in patients with PIPG, unlike in patients with SMART or ALERT syndrome [5,11]. When comparing the clinico-radiographic features of SMART and ALERT syndrome, Di Stefano et al. found that the latter was associated primarily with encephalopathic symptoms ranging from mild psychomotor slowing to severe vigilance impairment while MRI showed acute multifocal abnormalities in the subcortical and/or periventricular white matter characterized by punctuate or ring contrast enhancement [5]. Contrarily, SMART syndrome was associated with only mild encephalopathy and "focal" stroke-like deficits with MRI showing the unilateral cortical-subcortical area of hyperintensity and swelling on T2/FLAIR images, with prominent cortical enhancement in a multilobar and gyriform pattern [5].

To date, there are no standard treatment guidelines for SMART syndrome due to the rarity of cases and the lack of uniformity in the approach. However, several studies suggest that AEDs are a cornerstone of treatment. Valproate, carbamazepine, and LEV have been commonly used, with favorable outcomes particularly, in cases in which episodic neurological dysfunction is associated with seizures [3,14-16]. Additionally, partial or complete recovery has been reported to occur with pulse therapy of corticosteroids, which consists of 1 g IV/day for a five-day duration [1,6]. This was the case in our patient who responded favorably to IV methylprednisolone 1 g daily for a total of five days. Other reports have described the role of calcium-channel blockers in preventing migraine frequency and severity as well as the use of aspirin therapy (in treating "stroke-like" symptoms), although this is anecdotal [3,14,17]. Further understanding of the exact pathogenesis of SMART syndrome is needed to shed clarity on the role of these medications, if any, in treating this condition.

Similar to SMART syndrome, LMD is classically diagnosed via MRI and CSF cytology [18]. However, contrast enhancement is typically seen along cranial nerves, cerebellar folia, the spinal cord, or the cauda equina [18,19]. Enhancement of these structures was not seen on our patient’s MRI, and therefore a diagnosis of SMART syndrome was favored over LMD. In LMD, CSF analyses are often notable for lymphocytic pleocytosis, elevated protein, and decreased glucose representing the most frequently observed, but nonspecific findings [19]. However, false negative findings can sometimes be observed, and prompt further testing in the form of CSF circulating tumor cells and cell-free tumor DNA provide an opportunity for tumor analysis via liquid biopsies [18]. Unlike SMART syndrome, treatment of LMD consists of surgical and medical intervention in the form of palliative VPS; radiation (either craniospinal, whole-brain, or focal); immunotherapy; or intrathecal, systemic, or targeted chemotherapy [18,20].

The case illustrated here is characterized by headaches with new-onset seizures with subsequent status epilepticus that occurred several years after CSI. The patient’s history combined with radiotherapy and MRI evidence of leptomeningeal enhancement led to an initial concern for LMD. However, investigative studies including CSF analysis to rule out malignancy, infection, and inflammation were pivotal toward obtaining a correct and timely diagnosis. Likewise, involvement of both leptomeningeal and cortical gadolinium enhancement combined with an otherwise unremarkable CSF analysis made the diagnosis of LMD less likely and a diagnosis of SMART syndrome more favorable. The diagnosis was further supported by the fact that the patient’s symptoms and radiographic findings improved after the initiation of AED and steroid treatment. Knowledge of this potential diagnosis is important for avoiding unnecessary surgical biopsy procedures in patients with an otherwise self-limiting course following the initiation of medical management and treatment.

## Conclusions

SMART syndrome is a rare and underreported phenomenon that occurs years after radiation therapy. When evaluating MRI evidence of leptomeningeal and cortical enhancement, neurosurgeons should consider SMART syndrome in the differential diagnosis, especially when extensive workup rules out more common causes of this finding such as LMD. Proper identification of SMART syndrome can lead to timely treatment and improve clinical outcomes while avoiding the need for more invasive tests or diagnostic procedures.
